# Attenuated Ca^2+^ release in a mouse model of limb girdle muscular dystrophy 2A

**DOI:** 10.1186/s13395-016-0081-y

**Published:** 2016-02-24

**Authors:** Marino DiFranco, Irina Kramerova, Julio L. Vergara, Melissa Jan Spencer

**Affiliations:** Department of Neurology, David Geffen School of Medicine, University of California, Los Angeles, 90095 USA; Department of Physiology, David Geffen School of Medicine, University of California, Los Angeles, CA 90095 USA; Center for Duchenne Muscular Dystrophy at UCLA, 635 Charles E. Young Dr. South, NRB Rm. 401, Los Angeles, CA 90095 USA

**Keywords:** Calpain, Skeletal muscle, C3KO, Ca^2+^ release, Excitation-contraction coupling, RyR1, DHPR, Calpainopathy, Limb girdle muscular dystrophy, Calpain, Skeletal muscle, C3KO, Ca^2+^ release, Excitation-contraction coupling, RyR1, DHPR, Calpainopathy

## Abstract

**Background:**

Mutations in *CAPN3* cause limb girdle muscular dystrophy type 2A (LGMD2A), a progressive muscle wasting disease. CAPN3 is a non-lysosomal, Ca-dependent, muscle-specific proteinase. Ablation of CAPN3 (calpain-3 knockout (C3KO) mice) leads to reduced ryanodine receptor (RyR1) expression and abnormal Ca2+/calmodulin-dependent protein kinase II (Ca-CaMKII)-mediated signaling. We previously reported that Ca^2+^ release measured by fura2-FF imaging in response to single action potential stimulation was reduced in old C3KO mice; however, the use of field stimulation prevented investigation of the mechanisms underlying this impairment. Furthermore, our prior studies were conducted on older animals, whose muscles showed advanced muscular dystrophy, which prevented us from establishing whether impaired Ca^2+^ handling is an early feature of disease. In the current study, we sought to overcome these matters by studying single fibers isolated from young wild-type (WT) and C3KO mice using a low affinity calcium dye and high intracellular ethylene glycol-bis(2-aminoethylether)-*n*,*n*,*n*′,*n*′-tetraacetic acid (EGTA) to measure Ca^2+^ fluxes. Muscles were subjected to both current and voltage clamp conditions.

**Methods:**

Standard and confocal fluorescence microscopy was used to study Ca^2+^ release in single fibers enzymatically isolated from hind limb muscles of wild-type and C3KO mice. Two microelectrode amplifier and experiments were performed under current or voltage clamp conditions. Calcium concentration changes were detected with an impermeant low affinity dye in the presence of high EGTA intracellular concentrations, and fluxes were calculated with a single compartment model. Standard Western blotting analysis was used to measure the concentration of RyR1 and the *α* subunit of the dihydropyridine (αDHPR) receptors. Data are presented as mean ± SEM and compared with the Student’s test with significance set at *p* < 0.05.

**Results:**

We found that the peak value of Ca^2+^ fluxes elicited by single action potentials was significantly reduced by 15–20 % in C3KO fibers, but the kinetics was unaltered. Ca^2+^ release elicited by tetanic stimulation was also impaired in C3KO fibers. Confocal studies confirmed that Ca^2+^ release was similarly reduced in all triads of C3KO mice. Voltage clamp experiments revealed a normal voltage dependence of Ca^2+^ release in C3KO mice but reduced peak Ca^2+^ fluxes as with action potential stimulation. These findings concur with biochemical observations of reduced RyR1 and αDHPR levels in C3KO muscles and reduced mechanical output. Confocal studies revealed a similar decrease in Ca^2+^ release at all triads consistent with a homogenous reduction of functional voltage activated Ca^2+^ release sites.

**Conclusions:**

Overall, these results suggest that decreased Ca^2+^ release is an early defect in calpainopathy and may contribute to the observed reduction of CaMKII activation in C3KO mice.

## Background

Calpain 3 (CAPN3) is the muscle-specific member of a family of proteolytic enzymes commonly referred to as calpains [1]. Mutations in *CAPN3* lead to limb girdle muscular dystrophy type 2A (LGMD2A), a disease characterized by progressive muscle weakness and wasting [2]. CAPN3 is present in several skeletal muscle cell fractions including the cytosolic, the myofibrillar, and the membrane fraction [3]. In the membrane fraction, CAPN3 is highly concentrated at the triads, which are sites of contact between transverse tubules (T-tubules) and the terminal cisternae of the sarcoplasmic reticulum (SR). The interaction between voltage-sensitive calcium (Ca^2+^) channels (or dihydropyridine receptors (DHPR)) in the T-tubules with the SR’s Ca^2+^ release channels (or ryanodine receptors (RyR1)) is responsible for the excitation-contraction (E-C) coupling process [4].

The CAPN3 knockout mouse is used to model LGMD2A. Muscles of this mouse replicate many features of the LGMD2A phenotype such as reduced fiber diameter, mitochondrial abnormalities, and reduced muscle growth following a bout of atrophy [5–7]. In addition, there is loss of RyR1 from triad fractions, and concomitant loss of Ca^2+^/calmodulin-dependent protein kinase II (CaMKII) signaling.

We and others have demonstrated that CAPN3 can bind triad components such as RyR1 [3, 8], aldolase [3], and calsequestrin [8], but none of these associated proteins appear to be CAPN3 substrates [3]. On the contrary, we observed reductions in RyR1 levels in muscles from calpain-3 knockout (C3KO) mice and LGMD2A patients with decreased or absent CAPN3 [9]. Thus, CAPN3 appears to play a stabilizing (non-proteolytic) role for RyR1 at the triad, but the manner in which it accomplishes this task is still undefined.

Ca^2+^ triggers muscle contraction and regulates several signaling pathways that subsequently control downstream gene expression necessary for proper muscle remodeling, which in turn allow muscles to match gene expression with functional demands placed on them [10–12]. Such feedback leads to muscular plasticity allowing the muscle to meet metabolic demands and load-bearing capacity. We previously showed that isolated muscle fibers from C3KO release less Ca^2+^ upon activation than wild-type mice (C57BL) (WT) fibers but saw no difference in the rate of Ca^2+^ uptake [3]. These observations led us to hypothesize that impaired Ca^2+^ handling might cause deficits in downstream signaling pathways reliant on Ca^2+^. Subsequently, we showed abnormal CaMKII signaling, blunted gene expression, and impaired muscle adaptation in muscles lacking CAPN3 [9]. These studies revealed that the pathophysiological mechanisms underlying LGMD2A involve impaired Ca^2+^-mediated signaling and a weakened muscle adaptation response [9].

One important question in LGMD2A pathophysiology is whether the altered Ca^2+^ handling is an early pathological feature of calpainopathy. Our previous Ca^2+^ release studies were performed in aged C3KO mice (1 year old) using the permeant form of fura2-FF (fura2-FF AM, Teflabs, Texas, USA) in conjunction with video microscopy and a limited electrophysiological approach. While the affinity of this dye is appropriate to study fast Ca^2+^ release in skeletal muscle fibers, it is well known that permeant Ca^2+^ dyes can be internalized into organelles, thus confounding the interpretation of the data. In order to determine if intrinsic impairments in the Ca^2+^ release mechanisms are observed as an early feature of disease, here we used the impermeant form of the low affinity Ca^2+^ indicator Oregon Green BAPTA-5N (OGB-5N), in conjunction with large intracellular ethylene glycol-bis(2-aminoethylether)-*n*,*n*,*n*′,*n*′-tetraacetic acid (EGTA) concentrations (an approach that allows for accurate estimations of Ca^2+^ release fluxes in skeletal muscle fibers [13–15] and compared the properties of Ca^2+^ fluxes in single fibers of *flexor digitorum brevis* (FDB) and interosseous (IO) muscles between young adult C3KO and WT mice). The fibers were subjected to various stimulation paradigms under current and voltage clamp conditions, and OGB-5N fluorescence was measured using space-averaged and high-spatial-resolution detection approaches [16–18].

Overall, the results reveal that the magnitude of action potential which evoked Ca^2+^ release is indeed reduced in FDB fibers from young C3KO mice and that this impairment is consistent with a uniform reduction of the number of release sites per triad, since the kinetics and voltage dependence of the Ca^2+^ release process remains intact.

## Methods

### Animal handling and muscle fiber isolation

C57BL/6 and C3KO breeder mice were housed and bred in the UCLA vivarium in compliance with the regulations of the UCLA Department of Laboratory and Animal Medicine. All experimental protocols and use of animals were conducted in accordance with the National Institute of Health Guide for Care and Use of Laboratory Animals and approved by the UCLA Institutional Animal Care and Use for both strains; male mice of 9.5 to 16.5 weeks were used. Euthanasia was performed by deep isoflurane anesthesia followed by neck dislocation. FDB and interosseous muscles were dissected out, and single fibers were isolated by enzymatic digestion as reported previously [17, 18]. Several anatomical features (i.e., smooth surface, highly contrasted sarcomere striation and absence of kinks or contractures), as determined under bright field observation, were used as criteria to select fibers for the experiments. Morphological alterations (e.g., bifurcation and splitting) were not observed in fibers isolated from C3KO mice. All fibers used for the electrophysiological experiments were microscopically imaged, and their length and diameter were measured offline from the images. Fibers from C3KO animals had smaller diameters (by ~13 %) and length (by ~10 %) than those from controls; however, only the length differences were statistically significant. As a result, fibers from C3KO mutants had smaller surface areas (Table 1). Membrane capacitance, an indicator of both the surface-to-transverse-tubular-system (TTS)-membrane-area ratio and the intactness of the TTS was measured in most fibers under voltage clamp conditions. No significant difference was found between the membrane capacitance of WT and C3KO fibers (Table 1); furthermore, these measurements were similar to those previously reported [19–22].

**Table 1:** Geometrical parameters and membrane capacitance of isolated WT and C3KO fibers used for electrophysiological experiments

	Length (μm)		Diameter (μm)		Surface area (cm^2^ × 10^−4^)		Membrane capacitance (μF/cm^2^)	
	WT	C3KO	WT	C3KO	WT	C3KO	WT	C3KO
Mean (±SEM)	443.7 (±13.93)	402.1* (±7.25)	49.1 (±1.30)	43.1 (±0.6)	6.7 (±0.36)	5.6* (±0.14)	4.76 (±0.12)	4.69 (±0.09)
*N*	18	22	16	22	18	22	16	19

*Statistical significance (*p* < 0.05)

### Electrophysiology and calcium release measurements

Membrane potential was measured and controlled with a two microelectrode amplifier. Membrane potential was maintained at −90 mV in both current and voltage clamp experiments. Low resistance electrodes (~8 MΩ) were used. Both electrodes were filled with a solution (“internal solution”, see below) containing 250 μM of the low affinity calcium dye OGB-5N and a high concentration of EGTA (30 mM) semi-saturated with Ca^2+^ (2:1 [EGTA]:[Ca^2+^]). This mixture allowed for: (a) fixing the resting myoplasmic [Ca^2+^]concentration in all fibers to ~80 nM (a value close to the Kd of EGTA for Ca^2+^ at pH = 7.4), (b) recording fluorescence transients that approximate the Ca^2+^ release flux from the SR to the myoplasm, and (c) preventing contraction and the ensuing optical artifacts. After impalement of both electrodes, a 20-min period was allowed for equilibration between the pipette solution and the myoplasm. This assures that all measurements were performed in the presence of similar intracellular concentrations of the components of the internal solution (i.e., Ca^2+^ dye and EGTA). Calcium release was elicited by either action potentials (under current clamp conditions) or voltage pulses (under voltage clamp conditions). Action potentials were triggered by brief (0.5 ms) current pulses (15 % above threshold). Single current pulses or 1-s trains of current pulses (10, 20, 50, and 100 Hz) were used. The optical setup consisted of an inverted microscope equipped with (a) a standard epifluorescence attachment, (b) a custom-made confocal spot detection system, (c) a two-axis stage with 20 nm resolution scanning ability, (d) a CCD camera, and (e) two photodiode-based light detection systems [23–25].

Two fluorescence illumination/detection paradigms were used: wide-field (global) and confocal. Global illumination/detection allowed us to record space-averaged (multi-sarcomeric) Ca^2+^ release elicited by single and repetitive stimulation pulses (under current clamp conditions) and/or voltage pulses (voltage clamp conditions). For action potential-elicited Ca^2+^ release, fibers were rested 30 or 120 s, respectively, between single and repetitive current stimulations. For voltage clamp experiments, pulses of 20 ms in duration and amplitudes of −20 to 180 mV (with 10 mV increments) with respect to the resting potential were used, and fibers were rested 60 s between stimuli. Confocal illumination/detection was used to record spatially resolved (intra-sarcomeric) Ca^2+^ release elicited by single current pulses with 60 s rests between stimuli. For global detection, fluorescence was excited and recorded from a ~20-μm disk area at the center of the fiber, nearby the microelectrodes. For confocal recordings, a diffraction limited illumination spot (~0.8 μm FWHM) was used and the fiber was moved 200 nm stepwise [18, 25].

Ca^2+^ release signals are reported in terms of either dimensionless fluorescence (Δ*F*/*F*), or the underlying Ca^2+^ release fluxes (μM/ms), which are calculated using the single compartment model previously reported [15, 26] with modifications. The current model includes reaction equation for the following components: (a) 900 μM parvalbumin (Ca^2+^ binding: *k*_on_ = 0.025 μM ms^−1^, *k*_off_ = 0.7 ms^−1^; Mg^2+^ binding: *k*_on_ = 1.5 × 10^−5^ μM ms^−1^, *k*_off_ = 0.003 ms^−1^); (b) 240 μM troponin (Ca^2+^ binding: *k*_on_ = 0.15 μM ms^−1^, *k*_off_ = 0.45 ms^−1^); (c) 5 mM total adenosine 5′-triphosphate (ATP) (Mg^2+^ binding; *k*_on_ = 0.013 μM ms^−1^, *k*_off_ = 0.15 ms^−1^); (d) 30 mM EGTA (Ca^2+^ binding: *k*_on_ = 0.0105 μM ms^−1^; *k*_off_ = 0.00075 ms^−1^); (e) 250 μM OGB-5N (Ca^2+^ binding: *k*_on_ = 0.157 μM ms^−1^; *k*_off_ = 9.42 ms^−1^); (f) 5 mM total [Mg^2+^] (resting free [Mg^2+^] = 0.15 mM); and (g) 15 mM Ca^2+^ (resting free [Ca^2+^] = 0.075 μM).

Our model allows us to calculate action potential (AP)-evoked time-dependent changes in free [Ca^2+^] and [Mg^2+^]. We do not report these values since, as discussed elsewhere [15, 26], in fibers equilibrated with 15 mM EGTA, this is the dominant buffer and the free [Ca^2+^] transients are significantly depressed with respect to those under physiological conditions. The advantage of our approach is that movement is blocked, and [Ca^2+^] release fluxes can be accurately estimated [15, 26].

For current clamp experiments, we measured the amplitude of the action potentials and fluorescence transients and the depolarization at the end of the trains. For voltage clamp experiments, the peak and steady-state values of Ca^2+^ release transients were plotted as a function of the voltage; the data were fitted to Boltzmann functions of the form: Δ*F*/*F* = Δ*F*/*F*_max_ / (1 + exp((*V* − *V*_1/2_)/*k*)), where *k* and *V*_1/2_ are the slope (i.e., the e-fold change) and the midpoint of the distribution, respectively.

All experiments were performed at room temperature (21–22 °C).

### Solutions

Three solutions were used (mM): (a) internal solution: 75 aspartate, 5 ATP di-sodium, 5 phospho-creatine di-Tris, 5 reduced glutathione, 5 MgCl_2_, 30 EGTA, 15 Ca(OH)_2_, 20 3-(*n*-morpholino)propanesulfonic acid, 4-morpholinepropanesulfonic acid (MOPS), ph = 7.4 with KOH. The free [Mg^+2^] concentration in this solution was estimated (using published Mg^2+^ binding constants for ATP and parvalbumin) to be 0.15 mM. (b) Tyrode: 150 NaCl, 4 KCl, 2 CaCl_2_, 1 MgCl_2_, 10 MOPS, 10 glucose, pH = 7.4 with NaOH. (c) tetraethylammonium (TEA)-Cl: 150 TEA-OH, 10 CsCl, 2 CaCl_2_, 1 MgCl_2_, 10 MOPS, pH = 7.4 with HCl.

Osmolality of solutions was 300 ± 5 mOsmol/kg H_2_O. The internal solution was used in all experiments, Tyrode was used for current clamp experiments, and TEA-Cl was used for voltage clamp experiments. For voltage clamp experiments, tetrodotoxin (TTX) (200 nM), nifedipine (20 μM), 9-anthracene carboxylic acid (200 μM), and TEA (150 mM) were used to block sodium, calcium, chloride, and potassium currents, respectively.

### Muscle extract preparation for Western blotting

For Western blot analysis of total muscle extracts, FDB muscles were dissected and homogenized in a Dounce homogenizer in 30 volumes of reducing sample buffer (80 mM Tris, pH 6.8, 0.1 M dithiothreitol, 2 % SDS, and 10 % glycerol) with protease inhibitors cocktail (Sigma), boiled for 2 min and centrifuged at 12,000*g* ×10 min × 4 °C. The following antibodies were used for Western blot analyses: anti-calpain3 12A2 (Novocastra), anti-RyR1 (Thermo), anti-vinculin (Sigma), and anti-DHPRα (Thermo).

### Signal conditioning

Voltage, current, and optical data were filtered at 10, 5, and 2 kHz, respectively. Data were digitized at 30 μs/point using a National Instruments board (PCI-6221). Electrical stimulation, illumination, and data acquisition were under computer control, using a custom program written in LabView.

### Statistics

Data are reported as mean ± SEM. Means calculated from population data were compared using the Student’s *t* test. Significance was set at *p* < 0.05.

## Results

### Calcium release in response to single stimulation is smaller in C3KO fibers

**Fig. 1:**
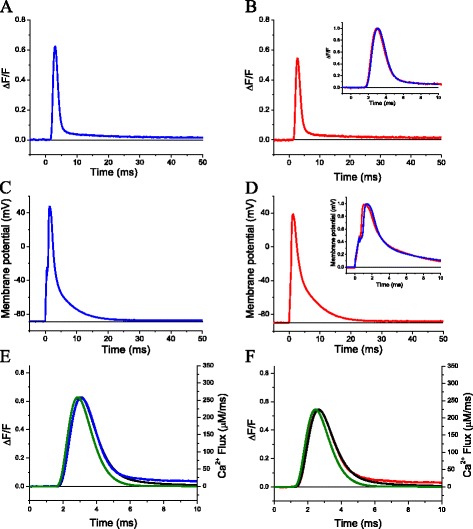
Ca^2+^ transients elicited by single action potentials in FDB fibers from WT and C3KO mice. **a** Representative single-sweep fluorescence transient recorded from a WT fiber. **c** The action potential triggering the fluorescence transient in (**a**). **b**, **d** The corresponding representative fluorescence transient and action potential recorded from a C3KO fiber. The peaks of the Δ*F*/*F* transients are 0.63 and 0.55 for the control and C3KO fibers, respectively. The fluorescence transients in (**a**) and (**b**) are shown normalized and superimposed in the *inset* of (**b**). The *inset* in (**d**) shows the superimposed normalized action potentials in (**b**) and (**d**. **e**, **f** The calculated Ca^2+^ fluxes (*green traces*, in μM/ms) underlying the fluorescence transients in (**a**) and (**b**), respectively. The *black traces* in (**e**) and (**f**) are the predicted Δ*F*/*F* transients (*left axis*), and the *blue and red traces* in (**e**) and (**f**) are the experimental Δ*F*/*F* transients in WT and C3KO, respectively. The *horizontal lines* represent zero Δ*F*/*F* for (**a**) and (**c**) (and *inset* in (**c**)) and −90 mV for (**b**) and (**d**)

In the current investigation, we performed a comprehensive comparison of the Ca^2+^ release dynamics between young C3KO and WT muscles using low affinity Ca^2+^ indicators that allow for study of calcium transients [3]. We first studied the Ca^2+^ release in fibers equilibrated with pipette solutions containing the low affinity calcium dye OGB-5N and a high concentration of free calcium buffer (EGTA, 15 mM). Typical electrical and fluorescence single sweep records are shown in Fig. 1. Figure 1a shows a Ca^2+^ transient, elicited by the AP shown in Fig. 1c, recorded from a WT fiber. It can be seen that after a short delay (of ~1.4 ms) from the start of the AP, the fluorescence reaches a peak in ~3 ms and decays exponentially afterwards. The full-duration at half-maximum (FDHM) of the transients (~2 ms) is slightly shorter than that of the AP itself (~2.3 ms, Fig. 1c). The transient decay can be approximated to a double exponential of the form Δ*F*/*F* = Δ*F*/*F*_0_ + *A*_1_*e*−^*t*/τ1^ + A_2_ e−^*t*/τ2^. The first (fast) exponential (*τ*_1_ = 1 ms, *A*_1_ = 0.63) accounts for ~95 % of the decay, while the second exponential (*τ*_2_ = 19 ms, *A*_2_ = 0.04) accounts for the remaining part of the transient’s decay phase. An example of the Ca^2+^ transient from C3KO fibers is shown in Fig. 1b. In this case, the amplitude of the Ca^2+^ transient (Δ*F*/*F* = 0.57) is smaller than that from the WT fiber (Δ*F*/*F* = 0.63, Fig. 1a); nevertheless, as shown in the scaled inset in Fig. 1b, when scaled and aligned, both transients are kinetically indistinguishable. As expected from this inset, the Ca^2+^ transient from the C3KO fiber could be fitted to a double exponential with time constants similar to those used for the WT fiber (*τ*_1_ = 1 ms, *τ*_1_ = 18 ms, *A*_1_ = 0.57, *A*_2_ = 0.03). In addition, the transients from WT and C3KO fibers reached their maximum values at similar times after the peak of the corresponding APs (1.62 and 1.53 ms, respectively). As observed in Fig. 1c, d, differences in the amplitude of the Ca^2+^ transients are not due to differences in the corresponding APs, since the electrical responses differ only slightly in amplitude. The similarity in kinetics is demonstrated in the inset of Fig. 1d, which shows both action potentials normalized and superimposed. Only a minor difference in the initial rate of rise of the APs, due to differences in stimulus pulse amplitude, can be noted. The Ca^2+^ signals in Fig. 1a, b were converted into Ca^2+^ fluxes and shown in Fig. 1e, f. In each example, the calculated flux (green traces) and the model simulated Δ*F*/*F* (black traces) are shown superimposed to the experimental records (blue and red traces for Fig. 1e, f, respectively). For each mouse strain, there is a close kinetic similarity between the experimental records and the calculated fluxes. In addition, the peak Ca^2+^ release flux in the C3KO fiber (224 μM/ms) is smaller than that from the WT fiber (260 μM/ms).

**Fig. 2:**
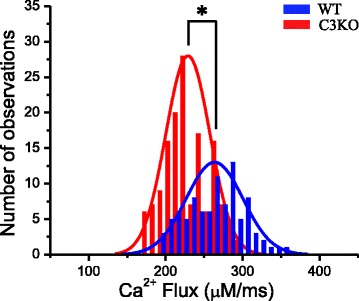
AP evoked Ca^2+^ flux is significantly smaller in C3KO fibers. Frequency distribution of peak Ca^2+^ fluxes from WT (*blue*) and C3KO muscle fibers (*red*). The lines of the corresponding colors are the Gaussian fits to the data. Data were obtained from four mice of each genotype. A total of 91 and 146 fluorescence transients recorded from 19 WT and 24 C3KO fibers, respectively, were used to calculate Ca^2+^ fluxes. The *asterisk* indicates statistical significance (*P* < 0.05)

In order to determine whether the apparent difference in Δ*F*/*F* and Ca^2+^ flux amplitudes (seen in Fig. 1) is statistically significant, we calculated the Ca^2+^ fluxes for the corresponding Δ*F*/*F* transients in a population of fibers obtained from four mice of each strain (Fig. 2). The peak Ca^2+^ fluxes for C3KO and WT fibers were fitted to single Gaussian distributions, represented by the blue and red lines, respectively. The distributions of Ca^2+^ fluxes were centered at 229 ± 3.9 (24 fibers, 146 records) and 265 ± 2.4 μM/ms (19 fibers, 91 records), for C3KO and WT fibers, respectively (Fig. 2b). This reduction in peak Ca^2+^ flux (~14 %) was statistically significant (*p* < 0.05).

### Frequency dependence of Ca^2+^ release in C3KO and WT fibers

**Fig. 3:**
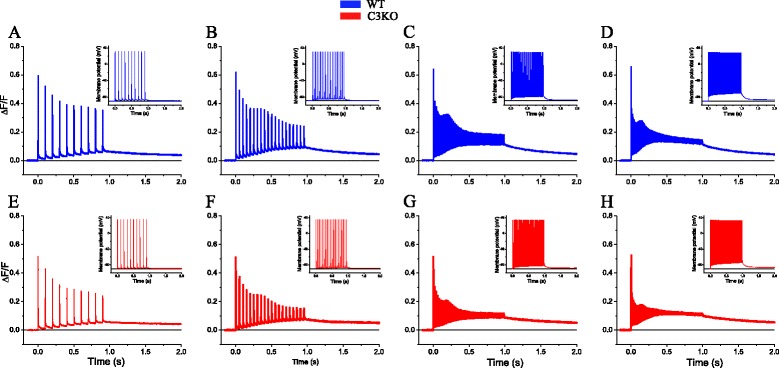
Ca^2+^ release in response to repetitive stimulation. Ca^2+^ transients recorded from WT (**a**–**d**) and C3KO fibers (**e**–**h**) were elicited by trains of pulses applied at 10 (**a**, **e**), 20 (**b**, **f**), 50 (**c**, **g**), and 100 Hz (**d**, **h**). Pulse and train durations were 0.5 ms and 1 s, respectively. The *inset* in each panel shows the corresponding electrical recordings. For all panels and insets, the *horizontal lines* represent zero Δ*F*/*F* and −90 mV, respectively

Muscle contraction in vivo is typically elicited by bursts of APs. In order to assess whether the depression in Ca^2+^ release that we observed with single pulse stimulation is also present in the responses to repetitive stimulation, we recorded fluorescence transients in response to trains of pulses applied at frequencies of 10, 20, 50, and 100 Hz (Fig. 3). The effect of frequency stimulation on the Ca^2+^ release of a WT fiber is shown in Fig. 3a–d. Under these conditions, the fluorescence transients display three salient features: (a) At all frequencies, the peak release in response to each stimulus becomes progressively (but non-monotonically) reduced along the trains; (b) the extent of reduction between the first and second transient and between the first and last transient are more pronounced at higher frequencies. Also, for all frequencies, there is an apparent increase in the peak release (i.e., resulting in a hump) that occurs at progressively earlier times from the beginning of the trains as the frequency of stimulation is increased. For example, for intermediate frequencies, the hump can be seen at ~400 ms for the train at 20 Hz, but at ~200 ms for the stimulation at 50 Hz. (c) Finally, the fluorescence during the inter-pulse interval increases during the trains and reaches values at the end of stimulation which increase as the frequency of stimulation increases. It is also apparent that, as a consequence of the peak decay and the increase in the inter-pulse values, the actual change in fluorescence elicited by each action potential also decreases during the trains. After the termination of the train, the fluorescence relaxes slowly to baseline.

C3KO fibers display a similar pattern of release as WT fibers in response to stimulation paradigms (Fig. 3e–h). As expected from single stimulation experiments (Fig. 1), the response to the first pulse of each train was smaller in C3KO fibers. In addition, for all frequencies, the maximum fluorescence change and the inter-stimulus fluorescence value at the end of the trains was also smaller in the C3KO fiber than those from the WT fiber (Fig. 3a–d). Moreover, the fluorescence decay after the trains was slower in C3KO fibers. While this was not further studied, it may result from a difference in SERCA activity between the two strains.

**Fig. 4:**
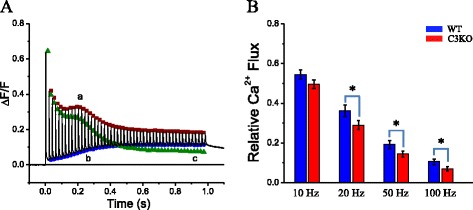
Depression of Ca^2+^ release during tetanic stimulation. **a** The calculation of the fluorescence transients elicited by each pulse during a 20-Hz train. The experimental record is represented by the *black trace*. The *brown* (**a**) and *blue traces* (**b**) are the envelopes to the peak and the inter-pulse fluorescence values along the train. The *green trace* (**c**) representing the amplitude of the fluorescence transients evoked by each action potential was obtained by subtracting (**b**) from (**a**). **b** Comparison of the mean ratio of Ca^2+^ fluxes in response to the first and last action potential of trains of 10, 20, 50, and 100 Hz in WT (*n* = 14) and C3KO (*n* = 18) fibers. *Asterisks* indicates statistical significance (*p* < 0.05)

In order to quantify the differences in the tetanic responses of WT and C3KO fibers, the peak (brown symbols) and inter-pulse values of every fluorescence transient along the trains (blue symbols) were approximated to envelope curves (traces a and b in Fig. 4a, respectively) drawn along the corresponding peak and pre-stimulus fluorescence values (traces a and b in Fig. 4a, respectively). By subtracting the envelope of the pre-stimulus values from that of the peak values, we obtained a curve that depicts the fluorescence changes evoked by each action potential, which are thought to closely track the underlying Ca^2+^ release (green symbols, trace c in Fig. 4a). An example of this analysis for the Ca^2+^ release in response to a 50-Hz train in a WT fiber is shown in Fig. 4a. As can be seen, the amplitude of the fluorescence transients decays along the trains, but the hump is less obvious; instead two phases of decay are seen: an early fast phase and a slower late phase. Although we do not have an explanation for this non-monotonic behavior of the peak fluorescence transients (and thus peak Ca^2+^ flux) in WT fibers, the same pattern was found in C3KO fibers (not shown). From this plot (Fig. 4, trace c), we calculated for every fiber the ratio between the amplitudes of the first and last fluorescence transient in every train. The results for this analysis for pooled data are shown in Fig. 4b. Except for the stimulation at 10 Hz, the ratio for C3KO fibers is significantly smaller than that for WT fibers (*P* < 0.05); this result suggests that Ca^2+^ release in C3KO fibers is not sustained as efficiently as in WT fibers.

### APs recorded from WT and C3KO fibers in response to tetanic stimulation

**Fig. 5:**
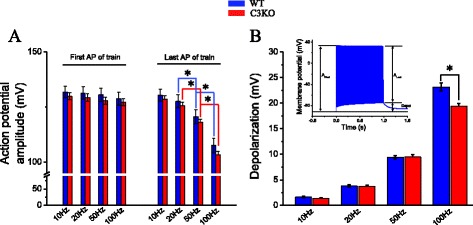
Electrical activity of WT and C3KO fibers in response to tetanic stimulation. **a** Mean amplitude (±SEM) of the first and last action potentials recorded from WT (*n* = 14) and C3KO fibers (*n* = 18) stimulated at 10, 20, 50, and 100 Hz. **b** Mean depolarization (±SEM) measured in WT and C3KO fibers at the end of trains of stimulation at 10, 20, 50, and 100 Hz. The *inset* demonstrates how the amplitude of the first and last action potentials and depolarization were measured in a record obtained in a C3KO fiber stimulated with a 100-Hz train of pulses (1 s). *Asterisks* indicate statistical significance (*p* < 0.05)

One possible cause for the progressive reduction in the magnitude of the Ca^2+^ release during repetitive stimulation in C3KO fibers could be that the APs are actually impaired. Although the insets in Fig. 3 suggest that this is not likely to be the case, we investigated this possibility quantitatively by measuring the amplitude of the first and last APs (as depicted in the inset of Fig. 5b) and the depolarization at the end of each train (Fig. 5b). No difference in the mean amplitude of the first AP was observed between the genotypes at all frequencies when tested independently (Fig. 5a and Table 2). The absence of a significant difference was still observed when the amplitudes of WT (130.66 ± 1.39; *n* = 54 records from 27 fibers; four mice) and C3KO (128.59 ± 0.83, 71 records from 27 fibers, four mice) were compared. In contrast, for both C3KO and WT muscle fibers, the mean amplitude of the last action potential (see inset in Fig. 5b) was significantly reduced as the stimulation frequency was increased. Nevertheless, when data from both strains were cross-compared, no difference was found; namely, both populations of fibers equally generate APs at all frequencies tested. Finally, for all the stimulation frequencies used, we compared the depolarization at the end of tetanic stimulation (Fig. 5b and inset). As expected, the basal depolarization at the end of the trains was more prominent at higher frequencies. It can be seen that the two populations of fibers behave similarly for frequencies below 100 Hz. At 100 Hz, we found that, on average, C3KO fibers depolarize less than WT fibers, while the amplitude of the corresponding APs is similar. Furthermore, when the duration of the APs was compared, no significant difference was found (not shown). Altogether, these observations imply that the reduction in Ca^2+^ release in C3KO fibers with respect to WT fibers does not result from electrical impairments (as illustrated in Figs. 3 and 4).

**Table 2:** Amplitude of the first and last action potentials (AP) elicited in WT and C3KO fibers by 1 s trains of pulses at various frequencies

	Action potential amplitude (mV)															
	10 Hz				20 Hz				50 Hz				100 Hz			
	First AP		Last AP		First AP		Last AP		First AP		Last AP		First AP		Last AP	
Strain	WT	C3KO	WT	C3KO	WT	C3KO	WT	C3KO	WT	C3KO	WT	C3KO	WT	C3KO	WT	C3KO
Mean (±SEM)	131.80 (±2.63)	129.84 (±1.59)	130.30 (±2.73)	128.58 (±1.57)	131.37 (±2.90)	129.30 (±1.71)	127.63 (±2.97)	125.58 (±1.54)	130.20 (±3.03)	127.93 (±1.66)	120.66 (±3.15)	118.12 (±1.35)	128.79 (±2.89)	127.28 (±1.63)	107.56 (±3.37)	103.34 (±1.55)
N	14	18	14	18	14	18	14	18	13	18	13	18	13	18	13	18

### The voltage dependence of Ca^2+^ release is not altered in C3KO

**Fig. 6:**
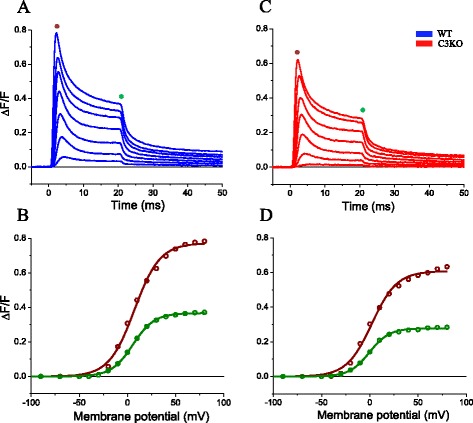
Ca^2+^ release from WT and C3KO fibers in response to voltage clamp pulses. **a** Fluorescence transients recorded in a WT fiber in response to depolarization to −10, 0, 10, 20, 30, 40, and 50 mV. **b** Voltage dependence of the peak and steady-state values of the fluorescence transients in (**a**). *Symbols* are the experimental values, and *lines* are Boltzmann fittings to the data. The fitting parameters were: Δ*F*/*F*_max_ = 0.77, *V*_1/2_ = 7 mV, *k* = 14 mV for peak values, and Δ*F*/*F*_max_ = 0.37, *V*_1/2_ = 6 mV, *k* = 11 mV for steady-state values. **c** Ca^2+^-dependent fluorescence transients recorded in a C3KO fiber in response to the same depolarizations as in (**a**). The *brown* and *green dots* in (**a**) and (**c**) indicated the time at which the peak and steady Δ*F*/*F* values were measured. **d** Voltage dependence of the peak and steady-state values Δ*F*/*F* transients in (**b**). *Symbols* are the experimental values, and *lines* are Boltzmann fittings to the data. The fitting parameters were: Δ*F*/*F*_max_ = 0.61, *V*_1/2_ = 2 mV, *k* = 13 mV for peak values, and Δ*F*/*F*_max_ = 0.28, *V*_1/2_ = 0 mV, *k* = 10.3 mV for steady-state values

Since the physiological trigger for Ca^2+^ release (i.e., the action potential) was not altered in C3KO, we investigated which step(s) of the E-C coupling process might be impaired in C3KO fibers. We first assessed whether the voltage dependence of the Ca^2+^ release was altered in the presence or absence of calpain 3. To this end, we measured fluorescence transients in response to voltage clamp pulses of various amplitudes. Since our model does not incorporate explicit equations of the voltage dependence of Ca^2+^ release, data are presented as Δ*F*/*F* transients. Fibers were rendered electrically passive by exchanging Tyrode by TEA-Cl. An experiment performed in a WT fiber is shown in Fig. 6a. Although the fiber was depolarized with 20 ms pulses applied from −90 mV in 10-mV steps, only transients in response to depolarizations to 0, 10, 20, 30, 40, 50, and 60 mV are shown. For every pulse, Ca2^+^-dependent fluorescence increases to an early peak and then decays to a steady-state value. Both the peak (indicated by the brown dot) and the steady-state values (indicated by the green dot) depend on the pulse amplitude, and both seem to saturate for large voltage pulses. Finally, the signals slowly relax to a baseline after the pulses end. To quantify the voltage dependence of the Ca^2+^ release, the peak and steady-state Δ*F*/*F* values of every transient were plotted as a function of the membrane potential, and the data were fitted to a Boltzmann function (Fig. 6b). In this fiber, peak and steady-state Δ*F*/*F* values were 0.77 and 0.37, respectively. The Boltzmann parameters were 7 and 6 mV (*V*_1/2_) and had slopes of 13 and 11 mV (*k*) for the peak and steady-state values, respectively. A similar experiment for a C3KO fiber is shown in Fig. 6c. The voltage dependence of the release from this fiber is shown in Fig. 6d; while the Δ*F*/*F* transients in the C3KO fiber has a similar profile as that of a WT fiber, the corresponding values for *V*_1/2_ (3 vs. 0 mV) and *k* (12 vs. 10 mV) values are smaller for C3KO.

**Fig. 7:**
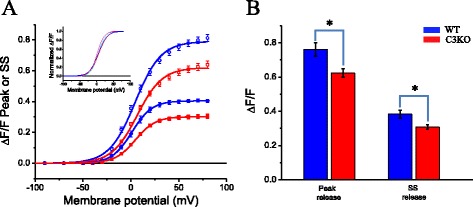
Voltage dependence of Ca^2+^ release in WT and C3KO fibers. **a** The *symbols* (and *bars*) are the mean values (±SEM) of peak (*open symbols*) and steady-state (*closed symbols*) of Δ*F*/*F* transients elicited by step depolarization in a population of fibers obtained from four WT mice (*n* = 10, *blue symbols and lines*) and four C3KO mice (*n* = 20, *red symbols and lines*). The *lines* are Boltzmann fittings to the data, obtained using the parameters in Table 3. The *inset* shows the same data normalized to their corresponding maxima. **b** Statistical analysis of mean maximal peak and maximal steady-state release (±SEM) for the same populations of data in (**a**). The *asterisk* indicates statistical significance (*p* < 0.05)

The peak and steady-state Δ*F*/*F* values in this example are 0.6 and 0.28, representing ~22 and ~25 % reductions in C3KO with respect to the WT values. The population analysis for the voltage dependence of Ca^2+^ release is summarized in Fig. 7 and Table 3. Figure 7a shows the superimposed voltage dependence for peak and steady-state of Δ*F*/*F* transients in populations of WT (*n* = 10) and C3KO (*n* = 20) fibers, each from two mice. The maximal peak Δ*F*/*F* for C3KO was 20 % smaller than those from WT fibers, while the maximal steady Δ*F*/*F* was reduced by 25 % in C3KO fibers. The statistical significance of such differences is shown in Fig. 7b. This difference was observed in the same populations of fibers that showed no difference in membrane capacitance (Table 1). Aside from these differences, no difference was found for *V*_1/2_ and *k* calculated from data acquired from WT and C3KO mice. The inset in Fig. 7a highlights the close superposition of corresponding data from both strains, demonstrating the similarity of all *V*_1/2_ and *k* values. In addition, data in Table 2 show the lack of statistical significance among corresponding slope and *V*_1/2_ values from WT and C3KO fibers.

**Table 3:** Parameters used to fit the voltage dependence of peak and steady-state Ca^2+^ release in WT and C3KO fibers to Boltzmann distributions

	Boltzmann parameters					
	(Δ*F*/*F*)_max_ (dimensionless)		*k* (mV)		*V*_1/2_ (mV)	
	WT	C3KO	WT	C3KO	WT	C3KO
Peak release (mean ± SEM)	0.76 (±0.04)	0.62* (±0.02)	13.43 (±0.36)	13.00 (±0.25)	4.31 (±1.38)	5.75 (±0.93)
Steady-state release (mean ± SEM)	0.38 (±0.02)	0.31* (±0.01)	10.21 (±0.38)	10.38 (±0.21)	1.24 (±1.52)	3.34 (±0.93)
*N*	10	20	10	20	10	20

*Statistical significance (*p* < 0.05)

The *k* and *V*_1/2_ parameters used to fit the voltage dependence of Ca^2+^ release from WT fibers (and C3KO fibers) reported here are in good agreement with those previously reported by us and others for FDB and interosseous fibers [20, 27–29].

### DHPR and RYR1 concentrations are reduced in C3KO muscles

**Fig. 8:**
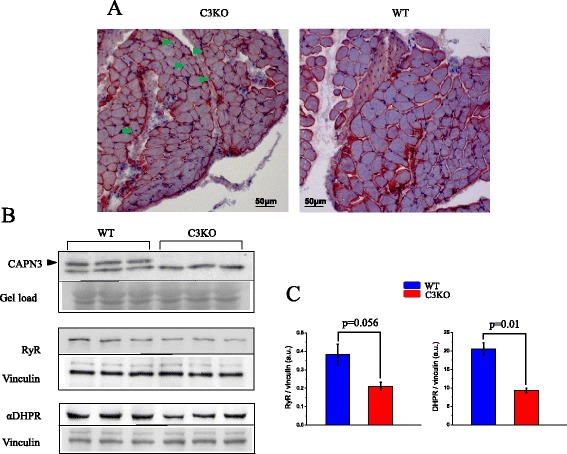
Expression of ryanodine and dihydropyridine receptors in WT and C3KO fibers. **a** Cross-sections of FDB muscles from WT and C3KO mice (HE). The *arrows* indicate non-peripheral nuclei. **b** Western blot analysis of CAPN3 (*upper row*), RyR1 (*middle row*), and αDHPR (*bottom row*) concentration in WT and C3KO muscles. *Arrowhead* indicates the CAPN3 band, which is absent in the C3KO muscles. **c** Quantitative comparison of the expression of αDHPR and RyR1. The *bars* are the means of densitometry measurements of triplicate data in (**b**)

We previously demonstrated that the concentration of RyR1 protein is reduced in muscles from 1 year old C3KO mice [9]. Since RyR1 and DHPR interact with a fixed stoichiometry at the triads, deviations due to altered expression of either receptor may have mechanistic implications for Ca^2+^ release; thus, we assessed the expression of both DHPR and RyR1 in FDB muscles from young mice used in the current investigation (Fig. 8). The FDB muscles from C3KO mice look histologically similar to age-matched WT FDB, as illustrated in Fig. 8a. C3KO muscles have a normal appearance barring that some fibers have mislocalized (non-peripheral) nuclei and that the cross-sectional area of the C3KO fibers is smaller. Western blot analysis in Fig. 8b demonstrates that the expression of both RyR1 and α subunit DHPR was significantly reduced in fibers lacking CAPN3. Densitometric analysis of the Western blots shows that RyR1 and αDHPR concentrations are reduced by 40 and 60 %, respectively, in C3KO (Fig. 8c). These results suggest a reduction in the number of Ca^2+^ release sites at the triads.

### Ca^2+^ release in C3KO is homogeneously reduced at all triads

While the biochemical data presented here are consistent with the above Ca^2+^ release studies, they do not allow us to predict alterations in the spatial distribution (i.e., the topology) of the voltage-dependent release sites among the triads.

**Fig. 9:**
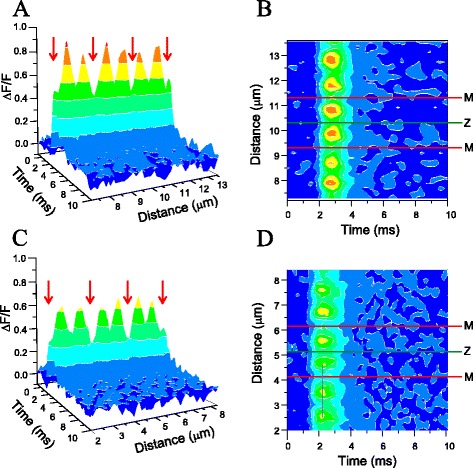
Ca^2+^ release domains in WT and C3KO fibers. **a** Tridimensional representation of Ca^2+^ release elicited by action potentials in a WT fiber. Confocal recordings were performed in positions separated by 200 nm along a ~6-μm stretch aligned parallel to the fiber long axis. **b** Intensity contour map representation of the data in (**a**). **c**, **d** The corresponding representations of confocal data recorded from a C3KO fiber. The same pseudocolor codification was used for all data. The *arrows* in (**a**) and (**c**) indicate the positions of M lines. The *red and green lines* in (**b**) and (**d**) indicate the positions of M and Z lines, respectively. The sarcomere length was 2.1 μm for both WT and C3KO fibers

To assess the topology of the predicted reduction in release sites (i.e., feet) per triad, we performed a confocal study of AP evoked Ca^2+^ release in C3KO fibers. Representative results of one fiber from each strain are shown in Fig. 9. Figure 9a is a 3D representation, spanning ~6 μm and 10 ms, of sub-sarcomeric Ca^2+^-dependent fluorescence changes recorded in a WT fiber. Six positions where the fluorescence reaches maximal values can be clearly identified; those sites correspond to the location of triads [16, 25].

**Fig. 10:**
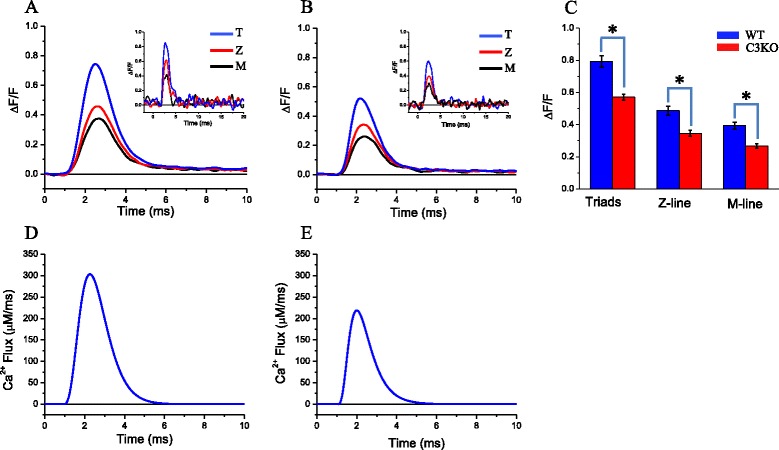
Comparison of Ca^2+^ transients at the triads and the M and the Z lines of WT and C3KO fibers. **a** Average Ca^2+^ transients at the triads (*blue trace*), the Z line (*red trace*), and the M line (*black trace*) calculated from a population of 12 WT fibers. **b** Equivalent data from a population of C3KO fibers (*n* = 6). The *insets* in (**a**) and (**b**) show representative single sweep Ca^2+^ transients recorded at the triad (*T*), Z line (*Z*), and M line (*M*) of a WT and a C3KO fiber, respectively. **c** Comparison of the average peak value of Δ*F*/*F* transients recorded at the triads, Z lines, and M lines. The *asterisks* indicate statistical significance (*p* < 0.05). **d**, **e** The average Ca^2+^ fluxes at the *T* sites (predicted by model simulations) for WT and C3KO fibers, respectively

Ca^2+^ diffusion away from these sites leads to the dynamic formation of spatiotemporal Ca^2+^ microdomains; they are centered at each transverse tubule (triad) and separated from each other by minima at the Z and M lines, the later indicated by arrows [17, 18]. The periodicity of peaks and valleys along the longitudinal axis of the fibers reveals a structurally organized and highly stereotyped Ca^2+^ release process. To provide a notion of the spatiotemporal formation of the Ca^2+^ microdomains, we present the same data in Fig. 9a, but as a contour map in Fig. 9b. This representation reveals that domains are short lived (~2 ms) and span ~1 μm each, as similarly reported elsewhere [17, 18]. A 3D reconstruction comparable to that in Fig. 9a, but for a C3KO fiber, is shown in Fig. 9b. As in the WT fiber, Ca^2+^ release is also stereotypically observed along the ~3 sarcomeres scanned, with the deepest valleys at the M lines (indicated by arrows) and maxima at the triads. Furthermore, consistent with the results using global illumination/detection described above, the peak amplitudes of the Ca^2+^ microdomains in the C3KO fiber are smaller than those in the WT fiber; notably, all the peaks are similarly reduced. Comparing the contour maps in Fig. 9b, d reveals the spatiotemporal similarity of the microdomains in both fibers. These patterns in Fig. 9a, b were seen in scans spanning 10–20 μm (not shown). In order to provide a more quantitative evaluation of the spatial distribution of Ca^2+^ release in C3KO and WT fibers, we studied the Ca^2+^ release from six WT and six C3KO fibers, obtained from two mice of each strain, and compared the Δ*F*/*F* transients and Ca^2+^ flux transients recorded at relevant positions along the sarcomere (i.e., the triads (T), the Z line (Z), and the M line (M)). To this end, we averaged the records at each position from 10 μm scans from each fiber and subsequently computed the mean values from all fibers of each strain (Fig. 10). Figure 10a illustrates that the average records from T, Z, and M sites from the WT population are clearly distinct from each other, as expected if the stereotyped pattern in Fig. 9a is preserved in all scans from all fibers. The inset in this figure shows typical single-sweep fluorescence transients, confocally recorded transients recorded at T, Z, and M sites of a WT fiber. The traces in Fig. 10a showcase the effective noise reduction achieved by averaging traces. Similarly, the position-dependent differences in amplitude evident from data in Fig. 9c persist after averaging data from a population of C3KO fibers as shown in Fig. 10b. For comparison, single sweep records from a C3KO fiber are shown in the inset of this figure. Notably, as suggested by the data in Fig. 9, peak values of averaged fluorescence transients recorded at T, Z, and M sites of C3KO fibers are smaller than those from corresponding records at equivalent positions in WT fibers. These differences are confirmed by the statistical analysis shown in Fig. 10c. We found that fluorescence transients at T, Z, and M sites from C3KO fibers are significantly smaller than the corresponding WT counterparts. Also, for each mouse strain, the changes at T, Z, and M sites are not uniform and are significantly different from each other (T > Z > M).

We finally used the fluorescence transients occurring at T locations (triads) to calculate the Ca^2+^ fluxes that would produce such transients. It should be noticed that these calculations allow estimating the actual maximal Ca^2+^ flux as it occurs at the sites of release (i.e., a voxel of ~1 μm^3^, as estimated from the *xy* and *z* resolutions of our confocal microscope [25]). The average Ca^2+^ flux transients are shown in Fig 10d, e for WT and C3KO data, respectively. For WT fibers (Fig. 10d), Ca^2+^ release flux at T locations (300 ± 13.5 μM/ms) is significantly larger than the 220 ± 7.4 μM/ms in C3KO fibers. Overall, these calculations demonstrate the Ca^2+^ release fluxes calculated at the triads are larger than those calculated from global recordings, which are space-averaged.

## Discussion

In the current study, we conducted a detailed analysis of Ca^2+^ release in a mouse model of LGMD2A and conclude that the absence of CAPN3 leads to impaired Ca^2+^ handling as an early feature of disease. We combined electrophysiological and optical methods to measure Ca^2+^-dependent fluorescence changes (Δ*F*/*F* transients) in isolated FDB fibers, loaded with a low affinity Ca^2+^ dye and high EGTA concentrations and maintained in current or voltage clamp conditions. Ca^2+^ fluxes were calculated from Δ*F*/*F* transients recorded using space-averaged (or global) and space-resolved (confocal) detection techniques.

We conducted the studies here on young mice because the phenotype of C3KO mice is progressive as the mice age [5]. Since our previous Ca^2+^ measurements were performed in aged mice, we were concerned that the defects in Ca^2+^ release might have been a consequence of structural abnormalities that accumulated over time. The data generated here showed that Ca^2+^ release was reduced and the expression level of both RyR and αDHPR were decreased in young C3KO mice compared to age-matched WT mice. Thus, these changes appear to be largely independent of the degenerative processes that occur in the pathogenesis of C3KO muscles during disease progression.

### Action potential evoked Ca^2+^ release is reduced in C3KO mice

In this study, we found that the Ca^2+^ fluxes elicited by single action potentials in C3KO fibers are significantly smaller than those from age-matched WT mice. These changes in amplitude are not accompanied by alterations in the kinetics of the flux transients. The limitation seen in Ca^2+^ release in response to single stimulation is more pronounced during repetitive stimulation, which is the physiological pattern used by the CNS to recruit mechanical output of muscles. Also, this attenuation in Ca^2+^ release increases with frequency, and this characteristic is expected to impact muscle mechanical output and fatigue resistance. A detailed comparative analysis of the electrophysiological data (Figs. 1, 2, 3, 4, and 5), obtained in all the regimes of stimulations used, demonstrated that differences in Ca^2+^ release in fibers from both genotypes of mice was not due to differences in the ability to evoke an AP.

Since electrode recordings only report membrane potential changes at the surface membrane, while Ca^2+^ release is controlled by the T-tubules, we measured the membrane capacitance of the fibers under experimentation. We found that alterations in Ca^2+^ flux in C3KO fibers was not due to differences in T-tubule-to-surface-membrane-area ratio, since the specific membrane capacitance of these fibers was not significantly different from that of WT fibers (Table 1). These data allow us to eliminate any gross structural alterations of the T-tubule system as an explanation for the data in the C3KO mice. In addition, the diameter of the limited number of C3KO fibers used for electrophysiological studies did not differ significantly from that of WT fibers (Table 1). Nevertheless, fiber diameter measurements in FDB cross-sections (Fig. 8) demonstrate that C3KO fibers are significantly smaller than WT ones, consistent with our published work and the known phenotype of LGMD2A patients. Overall, these results clearly indicate that one or more steps of the excitation contraction coupling process, beyond the action potential itself, is likely responsible for the blunted Ca^2+^ release in C3KO and are consistent with our observation of reduced RyR concentrations in the absence of CAPN3.

### Voltage dependence of the E-C coupling mechanism in C3KO

Since action potential generation and waveform are not altered in C3KO, we explored the possibility that the voltage dependence of the E-C coupling process is impaired in C3KO fibers. In this case, we evaluated the features of Δ*F*/*F* since our model does not incorporate explicit formalisms to predict the waveforms of Ca^2+^ release in response to voltage pulses. Nevertheless, the analysis above demonstrates that the use of either Δ*F*/*F* or Ca^2+^ flux transients leads to concurrent conclusions.

We found no voltage dependence alteration in either the peak or steady-state of Δ*F*/*F* transients (and thus in Ca^2+^ release) in response to voltage pulses. The slope and the midpoint voltage for activation of Ca^2+^ release from WT and C3KO are indistinguishable from each other. Instead, only a depression of both the peak and steady-state Ca^2+^ fluxes was found. Both values were reduced by ~20 % with respect to control values; a depression similar to that found in AP evoked Ca^2+^ fluxes. These results demonstrate that the voltage-sensing step of the E-C coupling process, i.e., the response of the αDHPR to membrane depolarization, is not altered in C3KO, while Ca^2+^ release is scaled down. These findings point towards possible alterations in the gain of the transduction process or the release process itself as the cause of the impaired Ca^2+^ handling.

### Ca^2+^ release is homogenously reduced in all triads of C3KO mice

Although it is not clear why the concentrations of both DHPR and the RyR1 are significantly reduced in C3KO mice (Fig. 9), our biochemical findings predict that there is a reduced number of release sites and/or an abnormal DHPR-RyR1 stoichiometry at the triads; this concurs with our functional data. One potential consequence of reduced expression of either, or both, RyR1 and αDHPR is an uneven distribution of functional triads along the sarcomeres. However, when we tested this possibility by means of a high temporal and spatial resolution method to detect submicron Ca^2+^-dependent fluorescence changes [18, 25], we found a stereotypical pattern of decreased Ca^2+^ release along the sarcomeres of C3KO with no sign of altered or failing triads along the stretches scanned (Figs. 9 and 10). Our confocal measurements concur with the capacitance measurements and suggest that the T-tubule system architecture is normal in C3KO mice. In addition, since the voltage dependence of the Ca^2+^ release is unaltered in C3KO mice, we speculate that the 1:4 RyR1:αDHPR stoichiometry to the release sites is also normal, and accordingly, our data can be explained if the number of (normal) release sites per triad is uniformly reduced along the sarcomeres.

### Ca^2+^ release and content of calpain, αDHPR, and RyR

One explanation for why the absence of CAPN3 leads to reductions of both DHPR and RyR1 may be related to the fact that the total amount of DHPR is tightly regulated in adult fibers [20] and is related to the level of RyR1 [30]. We have shown that calpain 3 maintains the RyR1 complex at the triad [3], thus the reduced RyR1 concentration is likely the explanation for the reduction in αDHPR.

The disparity between the reductions of Ca^2+^ fluxes (14–20 %) and the levels of both αDHPR (40 %) and RyR (60 %) is intriguing. In principle, the large reduction in αDHPR and RyR1 predicts a larger reduction in Ca^2+^ fluxes. Possible explanations are that not all the αDHPR and RyR1 participate in Ca^2+^ release in WT animals or alternatively that the gain of Ca^2+^ release is in fact increased in C3KO mice, thus partially compensating for the reduced number of release sites. Another possibility is that the impaired Ca^2+^ release at the triads of C3KO mice leads to a reduced Ca^2+^-dependent inactivation of the RyR, which partially compensates for the downregulation of RyR expression. Moreover, it is possible that the content of RyR1 and αDHPR is dependent on fiber diameter (i.e., smaller fibers are presumably more altered). Since the C3KO electrophysiological data were obtained from a population of relatively large (and uniform) diameter fibers, it is possible that their content of RyR1 and αDHPR is not as low as expected from biochemical data.

### Pathophysiological mechanisms in LGMD2A

In prior studies, we and others [7, 8] demonstrated that CAPN3 may play a structural role in maintaining the integrity of the triad-associated protein complex [3]. Importantly, we also showed that in LGMD2A patients, the concentration of RyR1 was decreased compared to healthy controls [9]. Decreased Ca^2+^ in skeletal muscle can affect several downstream signaling pathways that link muscle contractile activity with gene expression. This link is extremely important as it allows muscle to adapt as needed to the level and type of activity necessary, for example, by gradual transition of muscle fiber phenotype (i.e., fast or slow) or by switching the metabolic profile (i.e., oxidative vs. glycolytic metabolism). One such signaling pathway is mediated by CaMKII, which activates the transcription factor MEF2, to enhance gene expression of slow-type genes and facilitate the transition from fast to slow fiber phenotype. CaMKII signaling and several aspects of muscle adaptation are blunted in C3KO muscles [9] (and data not shown).

## Conclusions

These results suggest that decreased Ca^2+^ release may be an early defect in the pathogenic cascade of LGMD2A and that reduced Ca^2+^ release precedes impaired CaMKII signaling. The results are important because they establish key features of the pathogenic cascade in LGMD2A. Future studies are necessary to elucidate the precise role of CAPN3 at triads and its role in Ca^2+^ handling in skeletal muscle.

## Abbreviations

AP: action potential
ATP: adenosine 5′-triphosphate
C3KO: calpain-3 knockout
CaMKII: Ca^2+^/calmodulin-dependent protein kinase II
CAPN3: gene coding for calpain-3
FDHM: full-duration at half-maximum
EGTA: ethylene glycol-bis(2-aminoethylether)-*n*,*n*,*n*′,*n*′-tetraacetic acid
FDB: flexor digitorum brevis muscle
IO: interosseous muscles
LGMD2A: limb girdle muscular dystrophy type 2A
MOPS: 3-(*n*-Morpholino)propanesulfonic acid, 4-morpholinepropanesulfonic acid
OGB-5N: Oregon Green BAPTA-5N
RyR: ryadodine receptor (intracellular Ca^2+^ release channel)
SR: sarcoplasmic reticulum
TEA: tetraethylammonium
TTX: tetrodotoxin
WT: wild-type mice (C57BL)
αDHPR: alpha subunit of the dihydropyridine receptor or voltage-dependent Ca^2+^ channel (Cav1.1)
